# Automated Loss-of-Balance Event Identification in Older Adults at Risk of Falls during Real-World Walking Using Wearable Inertial Measurement Units

**DOI:** 10.3390/s21144661

**Published:** 2021-07-07

**Authors:** Jeremiah Hauth, Safa Jabri, Fahad Kamran, Eyoel W. Feleke, Kaleab Nigusie, Lauro V. Ojeda, Shirley Handelzalts, Linda Nyquist, Neil B. Alexander, Xun Huan, Jenna Wiens, Kathleen H. Sienko

**Affiliations:** 1 Department of Mechanical Engineering, University of Michigan, Ann Arbor, MI 48109, USA; hauthj@umich.edu (J.H.); safajb@umich.edu (S.J.); lojeda@umich.edu (L.V.O.); xhuan@umich.edu (X.H.); 2 Department of Computer Science and Engineering, University of Michigan, Ann Arbor, MI 48109, USA; fhdkmrn@umich.edu (F.K.); wiensj@umich.edu (J.W.); 3 Department of Software Engineering, School of Information Technology and Scientific Computing, Addis Ababa Institute of Technology, Addis Ababa 1000, Ethiopia; eyu2.wf@gmail.com (E.W.F.); mekonenkaleab0@gmail.com (K.N.); 4 Department of Physical Therapy, Ben-Gurion University, Beer Sheva 8400711, Israel; peregshir@gmail.com; 5 Division of Geriatric and Palliative Medicine, Department of Internal Medicine, University of Michigan, Ann Arbor, MI 48109, USA; lnyquist@med.umich.edu (L.N.); nalexand@med.umich.edu (N.B.A.); 6 VA Ann Arbor Healthcare System Geriatric Research Education and Clinical Center, Ann Arbor, MI 48105, USA

**Keywords:** loss of balance, activity recognition, body sensor networks, event detection, gait recognition, machine learning, wearable sensors

## Abstract

Loss-of-balance (LOB) events, such as trips and slips, are frequent among community-dwelling older adults and are an indicator of increased fall risk. In a preliminary study, eight community-dwelling older adults with a history of falls were asked to perform everyday tasks in the real world while donning a set of three inertial measurement sensors (IMUs) and report LOB events via a voice-recording device. Over 290 h of real-world kinematic data were collected and used to build and evaluate classification models to detect the occurrence of LOB events. Spatiotemporal gait metrics were calculated, and time stamps for when LOB events occurred were identified. Using these data and machine learning approaches, we built classifiers to detect LOB events. Through a leave-one-participant-out validation scheme, performance was assessed in terms of the area under the receiver operating characteristic curve (AUROC) and the area under the precision recall curve (AUPR). The best model achieved an AUROC ≥0.87 for every held-out participant and an AUPR 4-20 times the incidence rate of LOB events. Such models could be used to filter large datasets prior to manual classification by a trained healthcare provider. In this context, the models filtered out at least 65.7% of the data, while detecting ≥87.0% of events on average. Based on the demonstrated discriminative ability to separate LOBs and normal walking segments, such models could be applied retrospectively to track the occurrence of LOBs over an extended period of time.

## 1. Introduction

Falls place older adults at high risk of injury [1] and are among the leading causes of death [2] and non-fatal injury [3,4]. However, their occurrence is relatively rare [5]. Thus, capturing fall events to assess fall risk requires long periods (weeks to months) of monitoring. In contrast, losses of balance (LOBs) occur more frequently than falls and are associated with increased fall risk. Among older adults, most falls occur while walking and are often initiated by slips and trips [6]. LOBs can be defined as events “that would result in a fall if sufficient recovery mechanisms were not activated” [7]. Quantifying and characterizing these LOBs can be useful in assessing fall risk and in guiding interventions to prevent falls [8].

Most fall risk studies to date have been performed in clinical settings and involve a clinician evaluating intrinsic (e.g., fall history, sensory deficits, chronic illness) and functional (e.g., gait and standing balance performance) risk factors [9]. However, assessments in these controlled environments do not necessarily recreate real-world scenarios that result in falls. Thus, this assessment approach may not directly estimate real-world fall risk. Real-world LOBs are unanticipated and occur during various daily activities that may not be adequately recreated in laboratory settings [10,11,12]. For instance, while LOBs frequently occur during gait, gait characteristics among older adults have been shown to differ in the real world from those evaluated in laboratory settings [13,14]. Scripted LOBs typically exhibit stereotypical responses that are easier to identify, while real-world events possess more variable, “messier” characteristics. Real-world datasets are also typically large and thus more challenging to analyze.

Adaptation of clinical and laboratory fall risk assessment measures to estimate real world LOBs and falls are generally limited. At a high level, current LOB assessment approaches fall into two main categories: (1) methods relying on observations of functional gait assessment and the individual’s recollection of fall occurrence and (2) methods leveraging sensors to quantify and detect balance deficiencies, near-falls, and falls.

An individual’s recollection of fall or LOB occurrences may be convenient because the method does not require any equipment. However, assessments based on recollection of fall history present challenges in terms of their accuracy as they rely on the individual’s memory and subjective judgment [15]. Typical balance and gait observational functional assessments cannot be easily automated due to the need for human instruction and data collection. Sensors, however, can be used to measure kinematic data that can be indicative of balance decrements, LOBs, and falls while an individual performs conventional functional assessment tests (e.g., Romberg test and timed-up-and-go test), follows a scripted protocol [16,17], or receives a perturbation to simulate an LOB event [18]. While these approaches leverage technologies to extract objective measures of balance and fall risk, they do not currently capture the real-world fall risk of individuals, as measurements are only taken during scripted tasks. Wearable sensors offer an opportunity to apply these approaches to capture individuals’ gait and balance during daily activities. Accordingly, we used data from a sensor-based approach to quantify real-world LOB events during daily activities [19] and sought to apply machine learning (ML) to automate the identification of these events.

While ML has been successfully applied to data from wearable sensors to automatically detect falls [20,21], few studies have applied ML on datasets that included LOBs that did not result in a fall. Among the studies that have captured LOB events using IMU data, 21 or fewer LOB events were captured in each study and all events occurred during laboratory-based scripted protocols or treadmill gait trials [22,23]. While these studies report promising levels of accuracy, all were based on datasets collected under scripted and controlled laboratory settings. Alternative approaches involving ML methods for detecting LOB events have relied on other types of sensors, such as plantar force insoles, EEG sensors, radar, and vision-based methods [24,25,26,27]. IMUs offer multiple benefits over these alternative sensors. For example, IMUs are widely available, consume minimal power, are easily integrated into handheld/wearable electronics (e.g., phones, smartwatches), can measure multiple body segments, and are not affected by field-of-view restrictions, thus lending themselves to real-world applications outside of controlled settings.

As a preliminary step toward developing automated methods for detecting LOBs in large, real-world datasets (and ultimately, assessing real-world fall risk) in community-dwelling older adults, we used real-world data and a data-driven approach to develop, train, and test models capable of identifying real-world LOBs during daily activities in at-fall-risk older adults.

## 2. Materials and Methods

### 2.1. Overview

**Figure 1:**
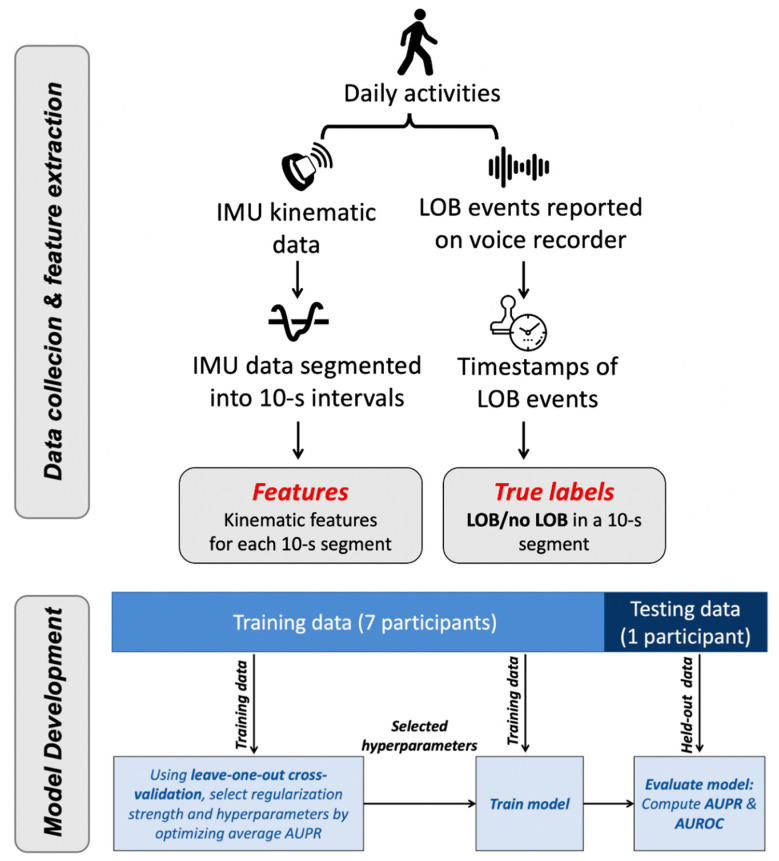
Overview of methods. Kinematic data were collected via wearable IMUs and used to extract relevant features. LOB events were reported and time-stamped to create true labels in the dataset. ML models were trained using leave-one-out cross-validation to automatically label LOB events based on kinematic features. Model performance was evaluated with respect to the AUPR and AUROC.

An overview of the data collection and data analysis flow is shown in Figure 1. We collected IMU data from at-fall-risk community-dwelling older adults. Participants were instructed to don a set of three IMU sensors, one on each foot and one on the lower trunk while they carried out their daily activities and reported the occurrence of LOBs in near-real time through a voice-recording device over a period of 2 weeks [11]. Using the IMU data with time-stamped LOB events, we extracted kinematic features representing participants’ movements throughout the day and applied ML techniques to build classifiers for automatically identifying LOB events. In contrast to other studies that solely included handpicked and pre-selected samples of data when validating models [22,23], we used the entirety of the data collected for any day during which there was an LOB event reported over the 2-weeks period for each participant. Finally, we evaluated the performance of these models by testing them on held-out data for one participant at a time.

### 2.2. Data Collection

#### 2.2.1. Study Cohort

Eight community-dwelling older adults (three females, five males, aged 69 to 82 years) with the ability to ambulate independently in the community, no physical activity contraindications, and at least one of the following inclusion criteria conferring fall risk participated in the study: a history of ≥2 falls in the past 6 mo, a history of an injurious fall in the past 6 mo, or self-reported balance difficulties [11]. Exclusion criteria included the use of a walker (which might alter the LOB pattern dramatically) or cognitive deficits (Montreal Cognitive Assessment score <24/30 [28]). All participants provided written informed consent. The study protocol was reviewed and approved by the University of Michigan Institutional Review Board (HUM00086479).

Study participants were equipped with a set of three wearable IMUs (Opal, APDM Inc., Portland, OR, USA; synchronized 128 Hz sampling). The IMUs measured linear accelerations and angular rates along the mediolateral (y-axis), anteroposterior (x-axis), and longitudinal (z-axis) axes. Participants were asked to wear the IMUs on their feet and lower back throughout the day, as they performed real-life, unscripted daily activities. Participants used a wrist-mounted, watch-sized voice recorder (Sansa Clip Zip 8 GB MP3 player, with a custom version of the RockBox open source firmware) to indicate when an LOB occurred and to describe the context of the event. The recorder was programmed to only record time- and date-stamped voice data for up to 2 min when a single button was pressed. The recorder, synchronized to the IMUs at the onset of each participant’s period of home wear, retained synchrony to within approximately 1 min per month and maintained battery power for the study duration.

#### 2.2.2. Outcome

Participants were instructed to report all LOBs (e.g., slips, trips) throughout the data collection period regardless of whether an LOB resulted in a fall, using a voice-recording device. During post-processing, the LOB events were cross-referenced and confirmed by a physical therapist based on foot velocity analysis and 3D animation kinematic reconstruction of the movements using the IMU signals collected [11].

### 2.3. Data Segmentation and Processing

#### 2.3.1. Data Segmentation

**Figure 2:**
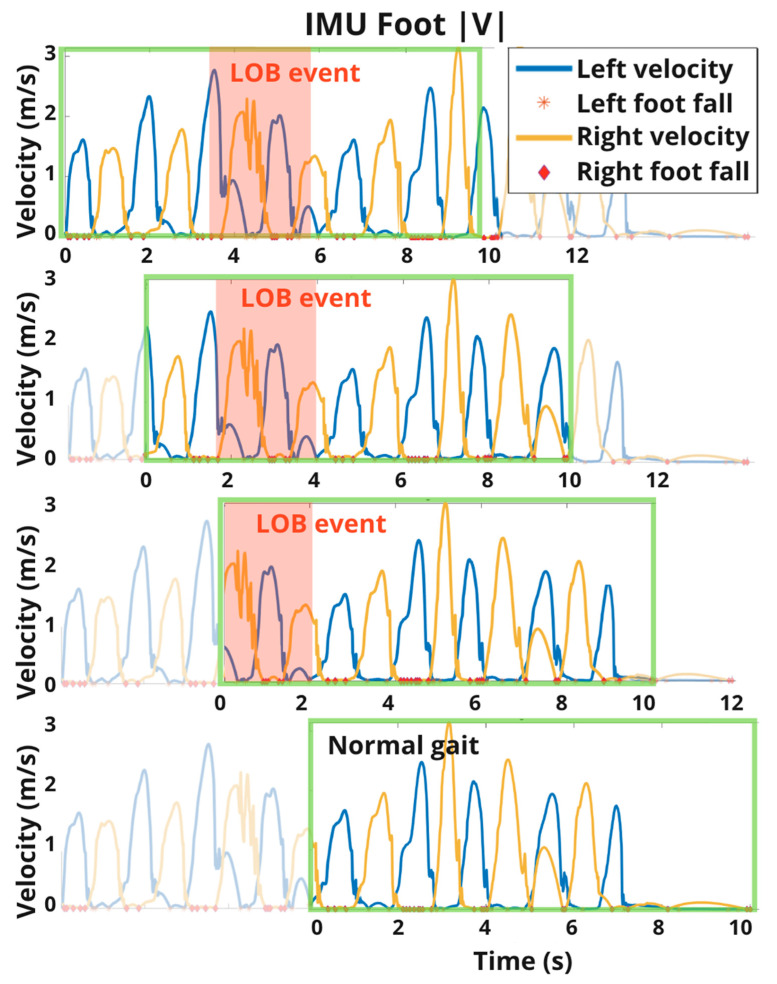
Time series data were segmented into 10 s segments with a sliding window with a stride of 2 s before extracting relevant gait metrics. A segment received the label LOB = 1 if it overlapped with any portion of the time-stamped LOB event. Otherwise, the segment received the label LOB = 0.

To establish the datasets for training and testing our ML models, IMU time series data for each day of data collection (a day of data collection lasted 11.4 ± 1.8 h) during which at least one LOB event was reported were segmented into short 10 s overlapping blocks such that each two consecutive blocks had a 2 s time difference (see Figure 2).

All LOB events in the dataset lasted less than 7 s, with most lasting 1–3 s. Thus, a 10 s segment with a 2 s stride ensured that every LOB occurred during at least one segment in its entirety. The sliding window segmentation approach did not rely on *a priori* knowledge of the event and ensured a variety of training and testing examples; e.g., some included LOB events near the beginning of these 10 s windows, some near the middle, and some near the end. The chosen window length could also be used for long LOB events that exceeded 8 s in duration (i.e., the overlap length between two consecutive windows), except that any single window would then only capture a portion of such a long event; we did not need to investigate these scenarios since we did not have any long LOB events in our data. For each 10 s window, we extracted the features (classifier input) and a binary LOB label (classifier output) described below in the Extraction of IMU-Derived Features and LOB Labels sections and subsequently built a ML model to map from the inputs to the outputs.

#### 2.3.2. Extraction of IMU-Derived Features (Inputs)

We used custom algorithms to integrate six-dimensional raw IMU sensor data (gyroscopes and accelerometers) to obtain foot orientation, velocity, and position using zero-velocity updates (ZUPT) to correct for sensor drift as described in Ojeda et al. [29,30]. Using foot trajectories, we determined individual strides and timing information and computed several common gait parameters (e.g., stride length, stride time) [31,32]. For each 10 s window, we extracted 32 features from the IMU data. Since the IMU data were collected in unscripted, real-world settings and included various activities other than gait, we first extracted a gait/no gait feature. We included a gait indicator feature since LOB events often arose from trips and slips during gait. A gait determination was assigned to each segment if it contained five or more strides longer than 0.1 m, where footfalls were detected based on foot velocities, assuming that the feet came to rest during the stance phase. This condition was defined based on reference gait parameter data for healthy participants ranging in age between 10 and 79 years [33]. The validity of this gait indicator was assessed visually to confirm that it correctly indicated walking segments. In addition to the gait indicator feature, we extracted 31 additional gait metrics corresponding to the total distance traveled, stride time, stride length, trunk angles, and angular velocities (Table 1).

#### 2.3.3. Extraction of LOB Labels (Outputs)

Each 10 s segment was assigned a positive LOB label (LOB = 1) if it overlapped with an identified LOB event either partially or completely. Otherwise, the segment was assigned a non-LOB label (LOB = 0).

### 2.4. Loss-of-Balance Classification Task

Using a leave-one-participant-out framework, we learned a mapping from the 32 IMU data features (inputs) to LOB labels (outputs) for each segment. Given a new segment of IMU data, this type of model could automatically estimate whether an LOB event had occurred.

#### 2.4.1. Model Architecture

Two model architectures were implemented and compared: (1) regularized logistic regression and (2) bi-directional long short-term memory (BiLSTM) [34,35].

**Figure 3:**
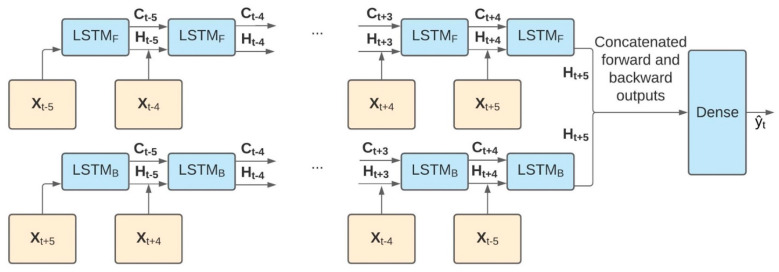
BiLSTM model architecture: feature vectors **X**_T_ shown in orange boxes at windows t−5 through t+5 and the predicted label ${\hat{y}}_{t}$ at time t shown in a green box. The BiLSTM model (parameters shown in blue boxes) used the five time steps both before and after the LOB. The memory of previous time steps was stored as **C**_T_, and predictions from previous time steps were stored as **H**_T_. Both of these information streams were passed to the next recurrent cell, either forward (F) or backward (B), and this accounted for contextual information at each time step. The linear model made use of the same features but in a flat vector (**X**_t−5_, **X**_t−4_, …, **X**_t+4_, **X**_t+5_), resulting in 374 features.

Regularized logistic regression models are commonly used for binary classification tasks as they are easy to implement and interpret and efficient to train. BiLSTM is a type of recurrent neural network well suited for sequence data and a close relative to the well-recognized long short-term memory (LSTM) network. An LSTM network processes sequential data in the forward direction and makes classification decisions using information only from preceding times. In contrast, a BiLSTM network processes sequential data in both the forward and reverse directions, capturing meaningful trends or indicators of an LOB that either succeeded or preceded the actual event. Features describing recent LOB recovery could be as useful as features describing an imminent LOB. Therefore, outputs from both the forward and backward BiLSTM layers were concatenated before going through a dense layer to make the final prediction. This model architecture was highly parameterized, and the degree of complexity was expressed in terms of cells, with the number of parameters being proportional to the number of cells. The complete pipeline is shown in Figure 3. This approach imposed that the automatic classification model account for up to 20 s of data following an LOB due to the 5-strided windows of 10 s that came after the earliest-possible LOB. Although this approach would make near-real-time LOB detection challenging, the delay associated with this approach did not negatively affect the model’s usability in the context of *a posteriori* fall risk assessment.

#### 2.4.2. Training Details

We set up our training and testing cases following a leave-one-participant-out scheme in order to assess the generalizability of our models across different participants. First, we created eight separate leave-one-participant-out *test* cases—that is, each test case had one of the eight participants’ data designated as the testing set, while the remaining seven were used for training and cross-validation. Then, in each of these sets of seven, leave-one-participant-out *cross-validation* was carried out to aid hyperparameter selection, where now one of the seven participants’ data were left out for cross-validation, while training was performed on the remaining six. For both testing and cross-validation, we split the data based on participants such that no participant appeared in both training and validation. Failure to do so could have resulted in the model simply learning to memorize individual participants, which would be of little clinical value.

Training was performed by minimizing the binary cross-entropy loss using the Adam optimizer [36] with a learning rate of 0.0001. The hyperparameters investigated included L1 and L2 regularization parameters for both logistic regression and BiLSTM models $\;(10^{-5},10^{-4},10^{-3},10^{-2},10^{-1}$) and the number of cells (2, 5, 10) of the BiLSTM model (i.e., the complexity of the model and how many parameters were learned). During training, we repeatedly up-sampled LOB events to aid with class imbalance. This data augmentation technique improved the class imbalance of LOB:non-LOB labels from approximately 1:1520 to 1:1 and helped prevent models from simply always predicting the majority class. BiLSTM models with the above settings were trained for 50 epochs, and the best hyperparameters were selected based on the lowest cross-validation loss. After cross-validation, our final models were trained with the best hyperparameters: L1 and L2 regularization of 0.01 and $10^{-5},$ respectively, for logistic regression, and L1 and L2 regularization of 0.1 and 0.001, respectively, for the BiLSTM with two cells.

#### 2.4.3. Evaluation Details

Since the distribution of classes was highly unbalanced (ratio of labels LOB:non-LOB was approximately 1:1520 windows in our case), an accuracy measure would be misleading (e.g., without any training, a constant classifier always predicting non-LOB would still achieve an accuracy of >99% for this study). Instead, we evaluated our models using the areas under the receiver operating characteristic curve (AUROC) and the areas under the precision recall curve (AUPR). The AUROC measures the performance trade-off between the true-positive and the false-positive classification rate. A random classifier based on the prevalence of the positive label would have an AUROC of 0.5, and a perfect classifier would have an AUROC of 1. The AUPR measures the performance of the classification precision (i.e., positive predictive value) relative to the classification recall (i.e., sensitivity). A random classifier would have an AUPR equal to the incidence of the true label, and a perfect classifier would have an AUPR of 1. For each test dataset, empirical confidence intervals were calculated using 200 bootstrapped samples.

In addition to reporting classification performance with respect to individual 10 s windows, we also reported overall data reduction, sensitivity, and precision at the event level for a 0.5 classification threshold. Overall data reduction was calculated by dividing the number of negative predictions by the number of windows in the dataset. At the event level, we defined a true positive as an LOB event for which our model predicted an LOB for at least one overlapping window (i.e., we had at least one alarm for that LOB event). Sensitivity was calculated by dividing the number of true positives by the number of LOB events. Precision was calculated by dividing true positives by all positive predictions. These metrics were chosen because they conveyed the model performance in a manner that was more directly useful to practitioners.

#### 2.4.4. Feature Importance

Furthermore, we performed a feature permutation importance analysis to determine whether any of the features in the dataset influenced the best model’s performance more than others. Deep neural networks, including BiLSTM, are not easy to interpret based on model parameters alone. We thus used permutation importance to test which feature, if swapped with the corresponding feature of different segments (with potentially different labels), yielded the greatest increase in model error. We implemented permutation importance measuring the change in both the AUROC and the AUPR when certain features were randomly shuffled between different segments, and then reported the final importance rank [37]. One limitation to this analysis was that permutation importance did not recognize feature redundancy. For example, if the same information was present among several correlated variables, the resulting feature importance might have been dampened because those same data remained intact in the correlated, non-permuted feature. We instead used the covariance matrix of the different covariates to identify groups of correlated features and permuted them together. Thus, rather than focusing on individual features, we opted for better information regarding the importance of highly independent feature groups [37]. By identifying block patterns in the feature covariance matrix, we split the 34 features into six groups: walked distance, stride length, stride time, trunk angles and angular velocity, swing and foot velocity, and the gait indicator. Variables in the same group were shuffled together during the permutation importance computation.

## 3. Results

Table 2 summarizes the number of days where at least one LOB event was observed, and the number of LOB events detected for each study participant. In total, 62 LOB events were identified based on voice recordings by the participants and verified via visual inspection of the signals by the study team.

The testing results for the final logistic regression model and the BiLSTM model are reported in Table 3 and Table 4.

For each test set, one study participant was held out, and we reported AUROC and AUPR scores to evaluate the performance of the models on each testing set. The relatively sparse occurrence of LOB events recorded in the data was reflected through the incidence rates reported in Table 4. The BiLSTM model outperformed the linear model in terms of the AUROC (*p* < 0.01), but performance benefits in terms of the AUPR were less significant (*p* = 0.07). In terms of the AUROC, BiLSTM performed consistently when testing across all participants, whereas the linear model occasionally outperformed BiLSTM by a small margin for some participants only to underperform BiLSTM by much wider margins for other participants.

For the BiLSTM model, overall data reduction, event-level sensitivity, and precision at a 0.5 threshold are reported in Table 5. The lowest average sensitivity score across all test cases was 87.0%, although most of them were greater than 90%. While these are the average sensitivity scores across multiple cross-validation trials, lower individual scores were observed for some trials, as reflected by the confidence intervals. We adopted the 0.5 threshold for this analysis as a reasonable demonstration, although it may be further optimized to achieve desirable goals (e.g., to reach a certain sensitivity or precision target).

For the BiLSTM model, the feature permutation importance of the feature groups included in the model varied based on the performance metric relative to which the ranking was calculated. Walked distance, stride length, and trunk angles and angular velocities were most important relative to the AUROC. Stride time, gait indicator, and stride length features were most important relative to the AUPR.

## 4. Discussion

Both BiLSTM and linear logistic regression models were able to successfully learn to identify LOB segments based on the kinematic dataset used, as reflected through AUROC scores exceeding 0.87 for the BiLSTM model and AUPR scores exceeding the incidence rate of LOBs for all test sets (for both models). The BiLSTM model outperformed the linear model based on test AUROC scores for all held-out participants, as shown in Table 3, and AUPR scores for most held-out participants, as shown in Table 4.

Reported feature permutation importance results reflected the kinematic characteristics of LOB events detected through the automatic classification models developed. The ranking of feature groups varied depending on the metric used to assess permutation importance. In the case of the AUROC, features relating to walked distance, stride length, and trunk rotations were of the most importance, whereas in the case of the AUPR, stride time, gait indicator, and stride length were most important. The observed variability may have indicated that the model learned from all of these feature groups as they all contributed to enhancing at least one of the performance scores calculated. These findings agreed with the characteristics of LOB events described in previous observational studies by Ojeda et al. [19] and Handelzalts et al. [11], where scenarios of compensatory trunk rotations and shorter strides were noted from wearable IMU data when participants slipped or tripped in real-world settings.

The AUPR scores computed through these analyses reflected a trade-off between precision and recall. Although the models learned to separate the two classes (LOB vs. non-LOB), they did not do so precisely; i.e., depending on the model, false-positive predictions occurred 100-500 times more often than true-positive predictions. The low precision results suggest that these models would not lend themselves to use-case scenarios involving the real-time detection or prediction of LOB events in real-world settings due to the high rate of false positives. Overall data reduction, event-level sensitivity, and precision, as reported in Table 5, further highlighted the models’ performance at identifying true LOB events as well as the challenges presented in terms of precision due to the high rate of false positives. The high sensitivity rates indicated that the models captured nearly all LOB events, while the overall data reduction values implied that significant portions of data (65.7–90.8%) were automatically filtered to greatly reduce the human effort of *a posteriori* analysis that targets only LOB events.

Based on the findings, the use of such models is better suited as an initial filter to reduce large datasets collected via wearables in real-world environments to smaller, more manageable datasets to support post-data collection analysis. As wearable sensing technology has evolved, there have been increased opportunities to transition from laboratory-based simulated LOB studies to real-world studies; however, barriers exist to fully leveraging potential real-world datasets, because current analysis methods require labor-intensive data segmentation approaches to identify likely LOB events [21,22,23]. The results from this study suggest that the developed models could eliminate up to 90% of data segments and facilitate more efficient search strategies for LOB events. Furthermore, due to the high sensitivity scores of these models, few LOBs would be eliminated as false negatives.

LOB events are inherently a rare occurrence during daily activities, although much more common than fall occurrence (only one fall was noted in this cohort). Due to the limited number of LOB events identified in the dataset, the models developed were built on a heavily skewed or unbalanced dataset, with an incidence of roughly 1:1520 positive-to-negative class labels. The up-sampling methods used were one attempt to partially address this issue. The use of a dataset with more incidents of LOBs should help improve the performance and generalizability of the models developed, despite the inherent imbalance between the two classes.

The results presented in this study underline the difference in difficulty between automatically detecting LOBs within laboratory versus real-world environments. In contrast to other studies that solely included handpicked and pre-selected samples of data when validating models [22,23], we used the entirety of the data collected from several days of daily activities that included LOB events for any given participant throughout the data collection period. In laboratory-based treadmill or over-ground studies involving scripted protocols, high (>0.85) sensitivity and specificity have been achieved through automatic binary classification, as reported by Weiss et al. [22] and Aziz et al. [23]. In the case of unscripted, real-world LOBs, however, we observed a significant drop in specificity due to the high rate of false positives detected by the models presented. Similar challenges have been reported when detecting falls, despite the more prominent kinematic features associated with falls in comparison with LOB events. Chaudhuri et al. [38] evaluated the real-world performance of a commercial fall detection wearable device whose manufacturer reported high specificity (>0.92) and sensitivity (>0.94) on laboratory-based validation data. In their findings, Chaudhuri et al. reported a much lower real-world sensitivity (<0.25) and low precision (<0.02). In another study, Palmerini et al. [39] attempted to address this challenge by training and testing a fall detection ML model on real-world datasets and were able to achieve higher sensitivity (>0.80) and precision (>0.53). The precision achieved, however, still fell short of the requirements of a real-world fall detection/alert system due to the high rate of false positives. These findings highlight the importance of training models on real-world datasets to optimize the performance of automatic event detection in the real world.

The primary limitations of this study were the limited number of LOB events and the lack of objective ground-truth data. This dataset included 62 LOB events, and the LOB event labels were based on participants’ voice recordings noting when a near-fall occurred and visual inspection of the kinematic data by study team members. Therefore, it is possible that some LOB events may have been unreported. Additional limitations included the small number of study participants; a larger number of study participants is likely needed to generalize our findings. In addition, by only including periods of data collection (days) during which LOB events were reported by study participants, the dataset used in this analysis did not capture the real incidence rate of LOB events over the 2-week data collection period.

This preliminary study demonstrated the use of an automatic LOB detection algorithm for real-world, unscripted daily-activity datasets. While the currently reported models showed good separability of the two examined classes (LOB and non-LOB), they presented challenges in terms of precision. Future work should incorporate datasets with additional LOB events in real-world settings with additional forms of ground-truth data.

## Figures and Tables

**Table 1:** Descriptive features extracted from kinematic IMU data.

Feature	Definition	Variables
Gait	Binary value (=1 if at least 5 strides of length >0.1 m)	IS GAIT (Binary)
Walked distance (m)	Total distance traveled in the horizontal plane	GAIT DISTANCE (Total)
Stride length (m)	Distance traveled in the horizontal plane between two consecutive footfalls of the same foot	SL MAX (maximum), SL MIN (minimum), SL MEAN (mean), SL MEDIAN (median), SL IQR (interquartile range), SL VAR (variance), SL RMS (root mean square)
Stride time (s)	Time elapsed between two consecutive footfalls of the same foot	ST MAX, ST MIN, ST MEAN, ST MEDIAN, ST IQR, ST VAR, ST RMS
Foot velocity (m/s)	Magnitude of foot velocities for both feet (left and right)	RF MAX VEL, RF MEAN VEL LF MAX VEL, LF MEAN VEL
Peak/Swing foot velocity (m/s)	Peak foot velocity magnitude for each foot (left and right) at every stride corresponding to swing phase	RS MAX VEL, RS MEAN VEL, RS MIN VEL LS MAX VEL, LS MEAN VEL, LS MIN VEL
Trunk angles (deg)	Angular sway in the pitch and roll directions	TRUNK RMS PITCH TRUNK RMS ROLL
Trunk angular velocities (deg/s)	Angular velocities in the pitch and roll directions	TW RMS PITCH TW RMS ROLL TW RANGE PITCH TW RANGE ROLL

**Table 2:** Number of LOB events for each participant.

Participant ID	Number of Days with Observed LOB Events	Number of Reported LOB Events
S 1	10	23
S 2	5	8
S 3	1	1
S 4	1	2
S 5	5	18
S 6	3	3
S 7	2	2
S 8	4	5
Total	31	62

**Table 3:** Test AUROC values for each held-out participant.

	Logistic Regression Model	BiLSTM Model
Participant ID	AUROC (95% CI)	AUROC (95% CI)
S 1	0.815 (0.659, 0.929)	**0.911** (0.887, 0.938)
S 2	0.802 (0.621, 0.937)	**0.902** (0.878, 0.927)
S 3	0.788 (0.627, 0.918)	**0.948** (0.916, 0.970)
S 4	0.808 (0.657, 0.938)	**0.942** (0.907, 0.977)
S 5	0.735 (0.575, 0.920)	**0.874** (0.843, 0.915)
S 6	0.816 (0.615, 0.934)	**0.906** (0.842, 0.950)
S 7	0.738 (0.550, 0.908)	**0.892** (0.859, 0.927)
S 8	0.778 (0.599, 0.936)	**0.946** (0.935, 0.958)

**Table 4:** Test AUPR values for each held-out participant.

		Logistic Regression Model	BiLSTM Model
Participant ID	Incidence Rate	AUPR (95% CI)	AUPR (95% CI)
S 1	0.066%	**0.006** (0.002, 0.009)	0.004 (0.003, 0.006)
S 2	0.061%	0.004 (0.001, 0.008)	0.004 (0.002, 0.009)
S 3	0.037%	0.004 (0.001, 0.008)	0.004 (0.002, 0.0057)
S 4	0.131%	0.004 (0.001, 0.008)	**0.030** (0.007, 0.101)
S 5	0.161%	0.003 (0.001, 0.006)	**0.005** (0.003, 0.006)
S 6	0.036%	0.005 (0.002, 0.009)	**0.011** (0.002, 0.041)
S 7	0.028%	0.003 (0.001, 0.006)	**0.005** (0.003, 0.008)
S 8	0.037%	0.004 (0.001, 0.008)	**0.005** (0.003, 0.010)

**Table 5:** Overall data reduction, sensitivity, and precision values for each held-out participant for the BiLSTM model.

Participant ID	Overall Data Reduction (95% CI)	Sensitivity (95% CI)	Precision (95% CI)
S 1	91.1% (89.7, 92.0)	87.0% (82.3, 90.9)	0.46% (0.37, 0.56)
S 2	68.0% (54.0, 72.6)	98.8% (95.8, 100)	0.18% (0.13, 0.23)
S 3	78.5% (62.0, 83.7)	100% (100, 100)	0.16% (0.08, 0.28)
S 4	84.7% (74.5, 86.9)	98.0% (93.4, 100)	0.81% (0.43, 1.21)
S 5	67.2% (52.3, 77.7)	98.7% (96.6, 99.7)	0.52% (0.44, 0.63)
S 6	85.5% (78.6, 89.6)	89.5% (83.1, 95.9)	0.21% (0.11, 0.31)
S 7	70.9% (55.8, 80.7)	100% (100, 100)	0.31% (0.23, 0.39)
S 8	91.0% (87.8, 92.9)	98.1% (93.2, 100)	0.39% (0.28, 0.54)

## Data Availability

The data presented in this study may be available on request from the corresponding author. The data are not publicly available due to ongoing analysis.
